# Pediatric Fecal Microbiota Harbor Diverse and Novel Antibiotic Resistance Genes

**DOI:** 10.1371/journal.pone.0078822

**Published:** 2013-11-13

**Authors:** Aimée M. Moore, Sanket Patel, Kevin J. Forsberg, Bin Wang, Gayle Bentley, Yasmin Razia, Xuan Qin, Phillip I. Tarr, Gautam Dantas

**Affiliations:** 1 Center for Genome Sciences and Systems Biology, Washington University School of Medicine, St. Louis, Missouri, United States of America; 2 Department of Pediatrics, Washington University School of Medicine, St. Louis, Missouri, United States of America; 3 Department of Pathology and Immunology, Washington University School of Medicine, St. Louis, Missouri, United States of America; 4 Division of Gastroenterology, Department of Pediatrics, Children’s Hospital and Regional Medical Center, Seattle, Washington, United States of America; 5 Department of Microbiology, Seattle Children’s Hospital, Seattle, Washington, United States of America; 6 Department of Laboratory Medicine, University of Washington, Seattle, Washington, United States of America; The University of Hong Kong, Hong Kong

## Abstract

Emerging antibiotic resistance threatens human health. Gut microbes are an epidemiologically important reservoir of resistance genes (resistome), yet prior studies indicate that the true diversity of gut-associated resistomes has been underestimated. To deeply characterize the pediatric gut-associated resistome, we created metagenomic recombinant libraries in an *Escherichia coli* host using fecal DNA from 22 healthy infants and children (most without recent antibiotic exposure), and performed functional selections for resistance to 18 antibiotics from eight drug classes. Resistance-conferring DNA fragments were sequenced (Illumina HiSeq 2000), and reads assembled and annotated with the PARFuMS computational pipeline. Resistance to 14 of the 18 antibiotics was found in stools of infants and children. Recovered genes included chloramphenicol acetyltransferases, drug-resistant dihydrofolate reductases, rRNA methyltransferases, transcriptional regulators, multidrug efflux pumps, and every major class of beta-lactamase, aminoglycoside-modifying enzyme, and tetracycline resistance protein. Many resistance-conferring sequences were mobilizable; some had low identity to any known organism, emphasizing cryptic organisms as potentially important resistance reservoirs. We functionally confirmed three novel resistance genes, including a 16S rRNA methylase conferring aminoglycoside resistance, and two tetracycline-resistance proteins nearly identical to a bifidobacterial MFS transporter (*B. longum s. longum JDM301*). We provide the first report to our knowledge of resistance to folate-synthesis inhibitors conferred by a predicted Nudix hydrolase (part of the folate synthesis pathway). This functional metagenomic survey of gut-associated resistomes, the largest of its kind to date, demonstrates that fecal resistomes of healthy children are far more diverse than previously suspected, that clinically relevant resistance genes are present even without recent selective antibiotic pressure in the human host, and that cryptic gut microbes are an important resistance reservoir. The observed transferability of gut-associated resistance genes to a gram-negative (*E. coli*) host also suggests that the potential for gut-associated resistomes to threaten human health by mediating antibiotic resistance in pathogens warrants further investigation.

## Introduction

Increasing antibiotic resistance in human pathogens threatens to render many bacterial infections untreatable [1]–[3]. Human commensal microbiota harbor numerous functional antibiotic resistance genes which, in aggregate, comprise the human gut-associated resistome [4], [5]. These genes can be exchanged among gastrointestinal microbes [6], including potential pathogens, particularly during host stress [7]. Therefore, characterizing the diversity and mobility of the commensal resistome is essential to understand the dissemination of multidrug resistance in hospitals and communities. The establishment of human gut-associated resistomes is ideally studied in the pediatric fecal microbiota. This microbial community is dynamic in early life: a newborn’s sterile gut is colonized immediately after birth, and the population structure then fluctuates rapidly for several years until it attains a composition that likely persists into adulthood [8]–[10]. Alterations to the microbiota during this critical developmental period may permanently alter both its phylogenetic composition and its associated resistome. Exposure to a fairly limited set of antibiotics (mostly beta-lactams, macrolides, and folate-synthesis inhibitors) is common in children [11]–[13], and resistance to beta-lactams, tetracyclines, sulfonamides, macrolides, and chloramphenicol has been reported in infant gut microbiota [14]–[17]. However, previous functional investigations of human fecal microbiota suggest that pediatric resistome diversity may have been significantly underestimated by culture- and PCR-based studies [5]. Moreover, though culture- and PCR-based studies may identify resistance genes and phenotypes, the association of resistance genes with mobile genetic elements, a crucial risk factor for dissemination of resistance, is not readily assessed with those methodologies.

Our experimental design was optimized to capture a maximally diverse sample of the pediatric gut resistome, to identify mobilizable resistance genes at greatest risk for dissemination, and to select for clinically-relevant and novel resistance genes. To deeply characterize the fecal resistome of 22 healthy pediatric clinic patients aged 1 month to 19 years, we coupled functional metagenomic selections with next-generation (Illumina platform) sequencing and a recently developed pipeline for high-throughput assembly, and annotation of functionally-selected DNA (PARFuMS, Parallel Annotation and Reassembly of Functional Metagenomic Selections) [18]. We sought to optimally represent the pediatric gut resistome by maximizing both the number of subjects included (22, an order of magnitude higher than prior functional metagenomic studies of gut-associated resistomes [5]) and the diversity of functional resistance screens (18 antibiotics representing eight drug classes). Because metagenomic (microbial community DNA) libraries created in a model Gram-negative (*Escherichia coli*) host models the potential transfer of resistance genes from native intestinal microbes to human pathogens, we were able to select for resistance genes with the most potential to confer resistance to human pathogens, regardless of the genes’ functions in their native hosts. This protocol also facilitates novel gene discovery because antibiotic resistance is detected by functional selection rather than by homology to previously described sequences. Finally, because resistance genes are identified on contigs 1–5 kb in length, we are able to identify resistance genes at greatest risk for dissemination by virtue of their close association with mobile genetic elements.

## Results

### Population Characteristics

There were no significant differences between infants and older children and adolescents in library size or clinical characteristics (Table 1). One child was exposed to antibiotics in the month before sample donation, and one infant’s stool yielded ciprofloxacin-resistant *E. coli* in a predecessor study [19].

**Table 1:** Clinical characteristics of fecal sample donors.

	Infant (< = 12 mo)			Child (>12 mo)
**N**	8			14
**Age (years)**				
Median	0.52			6.71
Range	0.09–1.02			2.18–19.62
**Sex**				
Female	4 *(50%)*			6 *(42*.*8%)*
Male	4 *(50%)*			8 *(57*.*1%)*
**Antibiotic exposure in the last month**				
Yes	0			1 (*7*.*1%)*
No	7 *(87*.*5%)*			13 *(92*.*8%)*
Unknown	1 *(12*.*5%)*			0
Antibiotic-resistant bacteria cultured from stool*				
Yes		1 *(12*.*5%)*	0	
No		7 *(87*.*5%)*	14 *(100%)*	
**Household contact taking antibiotics in the last month**				
Yes	1 *(12*.*5%)*			3 *(21*.*4%)*
No	7 *(87*.*5%)*			10 *(71*.*4%)*
Unknown	0			1 (*7*.*1%)*
**Diarrhea in the last year**				
Yes	3 *(37*.*5%)*			2 *(14*.*3%)*
No	5 *(62*.*5%)*			10 *(71*.*4%)*
Unknown	0			2 *(14*.*3%)*
**Metagenomic Library Size (GB)**				
Median	6.42			5.93
Range	0.65–17.2			0.02–26.45

Infant and child groups were compared using a two-tailed Fisher’s Exact Test for categorical data and the Wilcoxon rank-sum test for continuous data. There was no significant difference between infants and children for any clinical variable. Resistant bacteria were cultured and clinical data were collected in a previously published study [19].

### Antibiotic Resistance Phenotype

**Figure 1:**
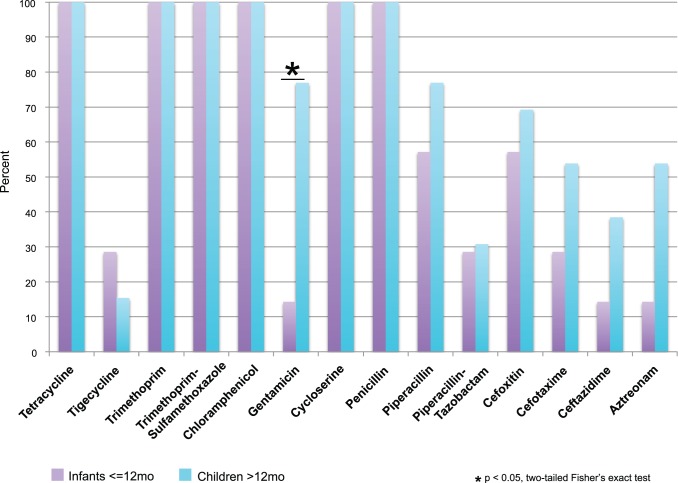
Antibiotic selections for which resistance was observed. The percent of libraries from infants (N = 8) and children and adolescents (N = 12) generating colonies resistant to 14 antibiotics is plotted. Two libraries that were <0.1 GB in size were excluded, as they did not have enough genetic diversity to accurately represent the resistance in their source metagenome. Not shown are four antibiotics for which no resistance was found: ciprofloxacin, meropenem, colistin, and cefepime.

Antibiotic resistance in the metagenomic libraries was stratified by subject age (infants < = 12 months, children and adolescents >12 months of age); with the sole exception of gentamicin, there was no significant difference between the groups in library resistance phenotype (Fig. 1). All libraries yielded *E. coli* transformants resistant to tetracycline, trimethoprim, trimethoprim-sulfamethoxazole, D-cycloserine, chloramphenicol, and penicillin. Decreased susceptibility was also found (though not uniformly) to aminoglycosides, glycylcyclines, and most beta-lactam classes.

### Resistance Genetics

There were 2489 contigs identified that bore at least one gene with predicted antibiotic resistance function. 840 of these belonged to multidrug resistance elements (defined as contigs bearing multiple resistance genes or a resistance gene and a transporter; 778 of these had multiple distinct antibiotic resistance genes, and 62 encoded resistance and transport proteins), 56 were single resistance genes syntenic with a mobile element (e.g. integron, transposon) and 14 belonged to multidrug-resistance elements syntenic with a mobile element (Fig. S1).

### Beta-lactam Resistance

**Figure 2:**
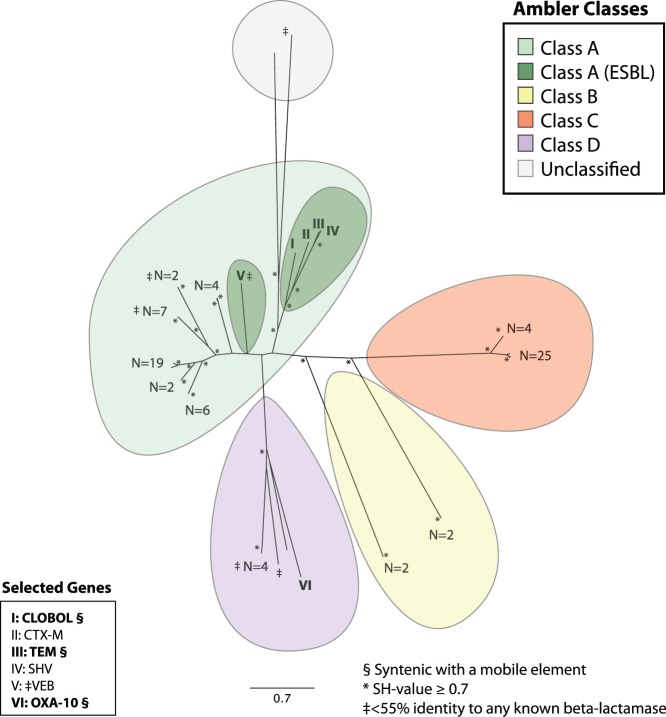
Unrooted approximate maximum-likelihood phylogenetic tree constructed using the predicted amino acid sequences of all beta-lactamases in the study set. Ambler classes are color-coded as indicated in the legend (upper right). At the terminus of each branch, the number of unique amino acid sequences in the terminal cluster is indicated. Branches with identity to extended-spectrum beta-lactamases (ESBLs) are numbered I–V and their putative ESBL classification is listed in the lower left legend of selected genes. A Class D beta-lactamase with high identity to a known OXA-10 is also numbered (VI) and included in the lower left legend. Genes included in the lower left legend that were syntenic with mobile genetic elements are bolded and marked with a §. Clusters of sequences with <55% ID to any known beta-lactamase are marked with a ‡. Nodes with a Shimodaira-Hasegawa (SH) value > = 0.7 are starred. The Shimodaira-Hasegawa score provides a measure of confidence in tree topology, with a maximum score (highest confidence) of 1.0 [52].

Genes from all four Ambler beta-lactamase classes were identified in infants, and classes A, C, and D were found in children and adolescents. Class A contained clusters of beta-lactamases similar to cephalosporinases previously identified in human fecal microbiota [5], and 12 unique proteins with <55% identity to any known beta-lactamase. A diverse set of predicted class A extended-spectrum beta-lactamases (ESBLs), including members of the TEM, SHV, CTX-M, and VEB protein families, were identified. Genes encoding members of the aforementioned Class A ESBL protein families were found in 4 of the 22 donors, which greatly exceeds previously reported rates of ESBL carriage in healthy children [20]. A fifth group of beta-lactamases, CLOBOL, was comprised of proteins with high amino acid identity to the putative beta-lactamase CLOBOL_04087 from *Clostridium boltae ATCC BAA-613* (GenBank Accession NZ_ABCC02000033.1). The function of CLOBOL has not been experimentally verified, and although it is not a known ESBL, it clustered with Class A ESBL protein families SHV, CTX-M, and TEM on an approximate maximum-likelihood phylogenetic tree (Fig. 2). CLOBOL was found in 8 of the 20 subjects (2 infants, 6 children <11 years old) and was closely associated with mobilization elements, which suggests significant dissemination of this poorly-understood beta-lactamase in this cohort of healthy pediatric clinic patients.

We found both pathogen-identical and novel putative beta-lactamase genes syntenic with mobile elements; these genes were often found in multiple genetic contexts, highlighting the dissemination of these genes within the gut microbiota of healthy children and adolescents. A beta-lactamase gene identical to one found on the drug-resistant *Neisseria gonorrheae* plasmid pJD4 (GenBank Accession NP_052173) [21] and with 99% identity to a known TEM ESBL (GenBank Accession AAL29436) [22], was also found in two genetic contexts with high nucleotide identity to pathogens extending beyond the gene boundaries: (i) on fragments with 98% identity to *E. coli* plasmids pAPEC-O2-R (GenBank Accession NC_006671) [23] and p838C-R1 (GenBank Accession HQ201416.1), and (ii) on fragments with 99% identity to the genome of the invasive pathogen *Haemophilus influenzae R2866* (GenBank Accession CP002277.1) [24]. A transposon-proximate Class D beta-lactamase identical to an OXA-10 beta-lactamase reported in the genome sequence of *Bacteroides sp. D22* (GenBank Accession NZ_GG774809) was found adjacent to a clindamycin resistance determinant (GenBank Accession NZ_GG705234), providing an example of disseminated resistance both to beta-lactams and a drug often used the treatment of beta-lactam-resistant Gram-positive infections [25].

### Aminoglycoside Resistance

**Figure 3:**
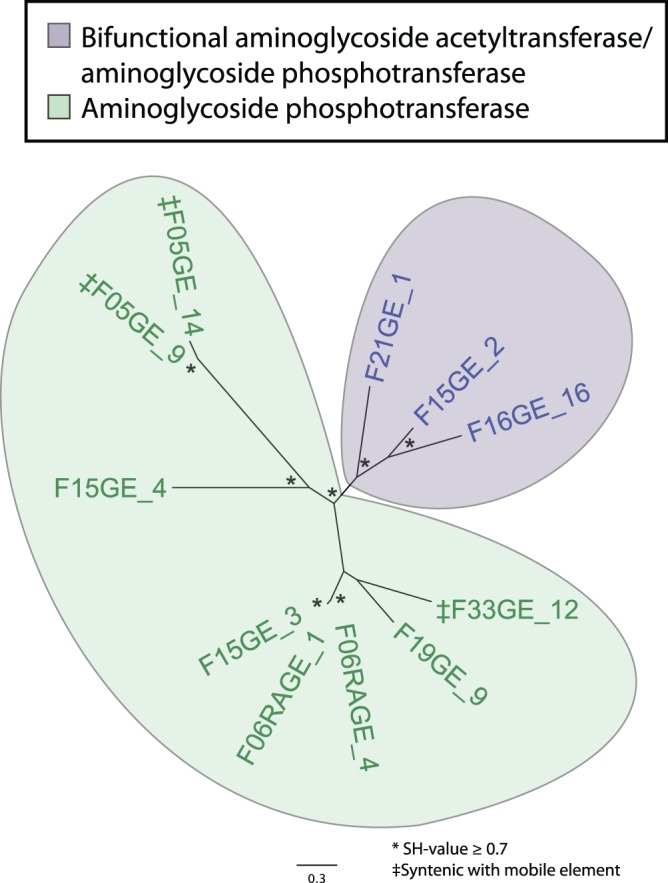
Unrooted approximate maximum-likelihood phylogenetic tree constructed using the predicted amino acid sequences of all aminoglycoside aminotransferases and phosphotransferases in the study set. Enzyme classes are color-coded as indicated in the legend. At the terminus of each branch, the name of the source contig is listed. Aminoglycoside phosphotransferases that were syntenic with aminoglycoside acetyltransferases are indicated with purple bold text and marked with a ‡. Genes syntenic with a mobile element are bolded and marked with a §. Nodes with a Shimodaira-Hasegawa (SH)-value > = 0.7 are starred.

Diverse predicted aminoglycoside acetyltransferases, phosphotransferases (Fig. 3), and adenylyltransferases were identified, as were several rRNA methylases (Table S6 in File S1), which presumably confer resistance via target protection. [26] A novel transposase-associated aminoglycoside phosphotransferase (F33GE_12) was only 52% identical to any known phosphotransferase, and nucleotide BLAST of the source contig against the NCBI nt and wgs databases revealed only low identity to several *Firmicutes* over one-third of the contig length, suggesting a cryptic source organism for this mobilizable resistance gene at risk for dissemination. Pathogen-homologous sequences were also found: one contig (F06RAGE_4) was identical to part of the *Clostridium difficile* 2007855 genome (GenBank Accession FN665654). The aminoglycoside-2″ phosphotransferase Ib encoded on this *C. difficile*-identical sequence was 99% identical to one identified in *Enterococcus faecium* (GenBank Accession 3HAM_A) [27].

We also characterized a novel 16S rRNA methylase with only 42% amino acid identity to an rRNA methylase from *Enterobacter cloacae* strain 115–824A (GenBank Accession JN968578). This rRNA methylase generates extreme resistance to gentamicin and amikacin, with clonal growth observed at 1024 ug/mL and 2048 ug/mL respectively (Table 2); clones remained susceptible to the closely related aminocyclitol spectinomycin (MIC 32 ug/mL for both clones and controls).

**Table 2:** Putative novel resistance genes.

Sample Name	Protein type	Antibiotic resistance (MIC ug/mL;control MIC, ug/mL)	Notes
19GentORF	16S rRNA methylase	Gentamicin (>1024; control 16), Amikacin(>2048; control 32)	42% amino acid identity to any known rRNA methylase
21MFSORF	MFS efflux protein	Tetracycline (32; control 4), Oxytetracycline(64; control 2)	99% amino acid identity to MFS transporter identified in *Bifidobacterium longum s. longum* JDM301
30MFSORF	MFS efflux protein	Tetracycline (32; control 4), Oxytetracycline(32; control 2)	98% amino acid identity to MFS transporter identified in *Bifidobacterium longum s. longum* JDM301
30NudixORF	NUDIX hydrolase	Trimethoprim (512; control 32), Trimethoprim-Sulfamethoxazole (32/608; control 2/38)	100% amino acid identity to Nudix hydrolase from *Lachnospiraceae* bacterium 7_1_58FAA.

These genes were not previously known to generate resistance, yet their resistance function was confirmed when the ORFs encoding them were cloned into *E. coli*.

### Chloramphenicol Resistance

Genes encoding predicted chloramphenicol acetyltransferases were commonly found: 22 metagenomic libraries contained 48 examples of these genes. Three chloramphenicol acetyltransferases syntenic with conjugative transfer proteins (TraG) or plasmid mobilization proteins (MobA/MobL) had low nucleotide identity to any known microbial genomes or plasmids in the NCBI nt or wgs databases, indicating that poorly characterized microbes might pose a risk for dissemination of chloramphenicol resistance genes (Table S7 in File S1). A fourth mobile resistance gene encoding a probable transposase-associated major facilitator superfamily (MFS) chloramphenicol transport protein, was identical over 98% of the total contig length to the *Corynebacterium resistens* plasmid pJA144188 (GenBank Accession NC_014167), which harbors nine other resistance genes and was recently sequenced from an isolate causing a bloodstream infection [28].

Several putative transcriptional regulators were identified, many belonging to the *marCRAB* locus regulating various microbial functions, including antibiotic resistance and stress response [29]. Constitutive expression of the *marA* transcriptional regulator and its homologs *SoxS* and *Rob* induce transferable antibiotic resistance in various microorganisms; all three were found in our samples [30], [31]. *MarA* and other genes in the *marCRAB* locus were also found in beta-lactam and tetracycline selections, consistent with the known activity of the *mar* regulon. Although these transcriptional regulators are only capable of generating antibiotic resistance in a host organism (such as *Salmonella enterica* or our model recipient *E. coli*) in which they can act to upregulate efflux pumps, their presence in healthy children is significant because they most likely reflect the presence of antibiotic-resistant *E. coli*, which, in addition to being a common component of the gut microflora, is also an important human pathogen.

### Tetracycline and Tigecycline Resistance

All three tetracycline resistance (*tet*) gene categories (ribosomal protection proteins, MFS and multi antimicrobial extrusion (MATE) transport proteins, and flavin-dependent monooxygenases) were identified (Table S8 in File S1), with multiple *tet* genes often found on the same DNA fragment. Several homologs of the *Bacteroides fragilis* conjugative transposon CTn341(GenBank Accession AY515263) [32] encoded TetQ ribosomal protection proteins syntenic with *rteA*, which regulates tetracycline-induced conjugative transposons [33]. One large (6 kb) contig encoding a TetW ribosomal protection protein and a TraG family conjugative transfer protein had very limited identity to sequences in the NCBI nt and wgs databases (homology to various *Clostridiales* over only half its length), implicating a heretofore uncharacterized organism as a source of mobilizable resistance.

In tigecycline selections, only sequences encoding flavin-dependent monooxygenases (TetX1 and TetX2) were found (Table S9 in File S1) on contigs homologous with the *ermF* region of the tetracycline-inducible *Bacteroides thetaiotaomicron* CTnDOT conjugative transposon (GenBank Accession AJ311171) [34], which encodes TetX1, TetX2 and an aminoglycoside adenylyltransferase. TetX modifies tigecycline, and TetX1 has been found in *Bacteroides* strains with increased tigecycline MICs [35].

Two putative MFS transporters not previously known to confer antibiotic resistance were confirmed to generate tetracycline resistance in the *E. coli* host. Each was >98% identical to an MFS transporter in *Bifidobacterium longum s. longum* JDM301; both conferred resistance to tetracycline (MIC 32 ug/mL) and oxytetracycline (MIC 32–64 ug/mL).

### Cycloserine Resistance

Contigs bearing genes that encoded D-ala-D-ala ligase were found to confer cycloserine resistance in all metagenomic libraries, possibly secondary to overexpression of plasmid-borne D-ala-D-ala ligase or D-alanine racemase by the *E. coli* host [36]. Notably, a contig (F30CY_50) bearing a D-ala-D-ala ligase, syntenic with a RecR recombination protein and a penicillin binding protein homologous to the penicillin-insensitive PBP2b, had a nearly identical (98%) protein sequence to several group D streptococcus (*Streptococcus bovis*) strains (Table S12 in File S1). Other contigs with D-ala-D-ala ligases syntenic with mobilization elements (F16CY_74, F18CY_75) had identity to uncultured organisms over <70% of the total fragment length (Table S1 in File S1).

### Trimethoprim and Trimethoprim-sulfamethoxazole Resistance

Genes encoding drug-resistant dihydrofolate reductase A were present in all libraries, and were frequently associated with thymidylate synthase and occasionally with aminoglycoside adenylyltransferases, MATE efflux proteins, and macrolide-lincosamide-streptogramin ABC transporters (Tables S10, S11 in File S1). Some contigs encoding dihydrofolate reductase and a mobilization element had high nucleotide identity to known commensals (e.g. F24TRSX_31, with >98% identity to *Streptococcus salivarius* (GenBank Accession CP002888.1)) [37]. Others were similar to pathogens: F30TRSX_13 had 99% identity to the *Klebsiella pneumoniae* plasmid pJIE137 (GenBank Accession EF219134), which is closely related to the p271a plasmid carrying the NDM-1 (New Delhi metallobetalactamase) that has been implicated in serious infections with carbapenem-resistant Gram-negative bacteria [38]. Several contigs with multiple resistance genes or mobilization elements were homologous to other microbes over only one-third of their total length (F11TRSX_80, F19TRSX_23), underscoring the potential importance of uncultured gut microbes harboring transferable resistance genes.

We also identified a putative novel mechanism of antibiotic resistance: a contig encoding a Nudix hydrolase identical to one reported in *Lachnospiraceae* bacterium 7_1_58FAA (GenBank accession NZ_ACTW01000031) was found to confer resistance to both trimethoprim (MIC 512 ug/mL trimethoprim; control MIC 32 ug/mL) and trimethoprim-sulfamethoxazole (MIC 32/608 ug/mL trimethoprim/sulfamethoxazole; control MIC 2/32 ug/mL) in the *E. coli* host. There are no prior reports of such resistance. Although this enzyme functions early in the pterin branch of the folate synthesis pathway [39], the exact mechanism of resistance is unknown.

## Discussion

Using high-throughput functional metagenomics, we captured a cohort of resistance genes from 22 healthy pediatric clinic patients representing greater molecular and mechanistic diversity than in any prior reports of antibiotic resistance in pediatric fecal microbiota [14]–[17]. Moreover, this population of healthy infants, children and adolescents harbored a diverse array resistance genes associated with mobilization elements and therefore poised for dissemination. The scale of this study, an order of magnitude larger than prior functional metagenomic studies of the human gut microbiota, was facilitated by the novel high-throughput coupling of next-generation sequencing with PARFuMS, a computational pipeline that assembles and annotates short-read sequence data from up to 200 functional selections in parallel, allowing dramatically increased throughput at <1% of the cost of Sanger-based methods [18] (details in File S1). Specifically, genes encoding all Ambler beta-lactamase classes were present, including pathogen-identical and novel proteins. Likewise, all major molecular mechanisms in the tetracycline-resistance family were represented. Chloramphenicol acetyltransferases were commonly found in this cohort, as were multiple-antibiotic-resistance transcriptional regulators (MarA and its homologs SoxS and Rob). All major classes of aminoglycoside-modifying enzymes were detected, as well as rRNA methyltransferases. Presumed drug-resistant dihydrofolate reductases and D-ala-D-ala ligases were present in every metagenomic library. Even some resistance genes for which antibiotic selections were not performed (e.g. macrolide-lincosamide-streptogramin resistance genes) were identified via proximity to other resistance genes. The use of an *E. coli* host organism facilitated detection of resistance genes with demonstrable ability to confer resistance to a model Gram-negative pathogen, and included many contigs with high sequence identity to widely varied organisms (commensals, pathogens, and both Gram-positive and –negative bacteria), representing a true cross-section of the fecal microbial community.

Observed antibiotic resistance phenotypes were similar in all members of this group of healthy pediatric clinic patients, and nearly every resistance gene class was found in both infant and child and adolescent age groups, strongly suggesting that the fecal resistome is established early in life. Although the limited clinical information associated with these archival samples precludes examining any association between antibiotic and other environmental exposures (e.g. family members, pets [10], [40]) and resistance phenotypes and genotypes, subject or family member exposure to several of the agents tested (tigecycline, D-cycloserine, tetracycline, and chloramphenicol) were extremely unlikely in this cohort. The capture of resistance genes against these agents, and the presence of resistance to tetracycline, chloramphenicol, and cycloserine in all libraries, suggests that, in some cases, the resistome is established independent of antibiotic selection in the host. In particular, elevated tigecycline MICs in our *E. coli* host were found in several libraries even though the fecal samples were collected before this agent was introduced [41]. Decreased tigecycline susceptibility was conferred by TetX1 and TetX2, which likely encodes a tetracycline-degrading enzyme similar to tetX; this presumably conferred decreased susceptibility to tigecycline due to structural similarities between tetracycline and tigecycline. These findings suggest a potential role for functional metagenomics in predicting emerging resistance to new antibiotics and improving surveillance and control of multidrug-resistant organisms.

The pediatric fecal resistome is potentially accessible to pathogens, so resistance genes associated with mobile elements deserve special attention due to their increased potential for dissemination. While some familiar mobile resistance genes were identified, such as the TEM beta-lactamase associated with *E. coli* R1 plasmids and *tet* family genes associated with *Bacteroides* conjugative transposons, we also identified mobilizable resistance genes on contigs with little identity to known microbial genomes or mobile genetic elements archived in the NCBI nt and wgs databases in every class of antibiotics assayed. This demonstrates that poorly characterized uncultivated organisms in the gut microbiota are important sources of resistance genes, even to rarely used antibiotics. The detection of chloramphenicol resistance in every library was especially surprising; prior culture-based studies of pediatric fecal microbiota detect chloramphenicol resistance less frequently than resistance to other agents [14]–[17], [42]. The factors underlying the common chloramphenicol resistance in the uncultured fecal microbiota are unclear, although selective pressures in the environment such as agricultural use of chloramphenicol [43], or vestigial resistance from an era when chloramphenicol was more widely used in humans, are plausible. Clearly, the pediatric fecal microbiota must be further characterized as an important reservoir of mobilizable resistance against common and rarely used antibiotics, particularly if the rise of multidrug-resistant pathogens spurs reintroduction of rarely used agents [44].

Novel resistance mechanisms encoded by contigs nearly identical to *Bifidobacterial* genomes illustrate the utility expressing genes in a model Gram-negative organism to identify potential interactions between probiotic and pathogenic gut microbes. Although many safety assessments of food-grade and probiotic organisms have been performed [45], the omission of functional selections in these experiments might have underestimated the potential of these organisms to act as resistance gene reservoirs. For instance, we show that two proteins >98% identical to MFS transporters from *B. longum s. longum JDM 301* generated high-level tetracycline and oxytetracycline resistance in *E. coli*, yet a detailed computational analysis of this strain did not associate these transporters with tetracycline resistance [46]. Although these results must be interpreted with caution, as the risk of horizontal gene transfer from probiotic to pathogen in the absence of a mobile genetic element is low, this example of a widely-used food-grade organism with the potential to donate high-level antibiotic resistance to a pathogenic host, regardless of the gene’s function in its native context illustrates that an organism’s potential to act as a reservoir of resistance genes is best assayed using functional metagenomics, a key complement to culture-based and *in-silico* approaches.

Finally, we report trimethoprim-sulfamethoxazole resistance conferred by a contig encoding a putative Nudix hydrolase, which depyrophosphorylates dihydroneopterin triphosphate in the pterin branch of the folate synthesis pathway [39]. The precise resistance mechanism is unclear: Nudix hydrolase acts far upstream of dihydrofolate reductase or dihydropteroate synthase, the respective targets of trimethoprim and sulfamethoxazole that have previously described drug-resistant variants [47]. Further research is needed to characterize this probable new resistance mechanism.

Do occult resistance genes in rarely studied commensal organisms threaten human health, or are they destined to remain sequestered in host bacteria unlikely to cause infections? Although our laboratory-designed transfer of resistance genes to the model recipient *E. coli* does not prove that such transfer occurs naturally, the ease of transferring resistance phenotypes to a model recipient closely related to many human pathogens is concerning. While current data are insufficient to assess the risk of commensals transferring resistance alleles to pathogens, the presence of functional resistance genes in healthy children that are more diverse than previously suspected and include a mobilizable resistance reservoir against rarely used antibiotics, is worrisome. Our identification of tigecycline resistance in fecal samples collected before introduction of that drug, of unanticipated resistance function of proteins (e.g. MFS transporter in *Bifidobacterium longum*), and of a putative novel resistance mechanism (Nudix hydrolase) demonstrate the potential for functional metagenomics to detect new patterns of resistance, and suggest that increased surveillance for resistance genes in commercial organisms (i.e. probiotics) and in human microbial communities, may be warranted. Together, these results underscore the importance of functional metagenomics as a complement to culture-based, PCR, and computational resistance assays, and illustrate its promise as a powerful tool for illuminating fecal resistome diversity and identifying emergent resistance trends.

## Methods

### Subjects and Specimens

22 deidentified stools archived at −80°C since collection for a study of fluoroquinolone resistance in healthy pediatric clinic patients [19] were selected for investigation. Fecal donors were infants, children, and adolescents ranging in age from one month to 19 years (Table S1 in File S1). Samples were randomly selected within defined age brackets. To ensure that the critical first year of life was adequately represented, eight samples were selected from infants < = 12 months of age. As these were deidentified archival specimens previously described in a prior publication [19], they were not considered to constitute human specimens per NIH regulations, and thus did not require additional IRB review.

### Metagenomic Library Construction and Functional Selection

Metagenomic libraries were constructed and screened as described [48] (details in File S1). Briefly, DNA was separated from 50–100 mg of frozen stool from each subject using bead-beating and phenol:chloroform extraction, sheared into 2–5 kb fragments, ligated into expression vector pZE21 [49], and electroporated into *E. coli* MegaX DH10B T1R (Invitrogen). These metagenomic libraries were screened for resistance to 18 antibiotics on Mueller-Hinton (MH) agar plates (Table S3 in File S1).

Functionally-selected metagenomic fragments were PCR-amplified, sheared into ∼160 bp fragments, ligated to adapters indexed with unique 7 bp nucleotide tags, and sequenced on the Illumina HiSeq 2000 platform. The SRA accession number for the raw reads is SRP029439. Reads were assigned based on their index tag (up to 96 selections sequenced per lane) before assembly into full-length metagenomic fragments and functional annotation using PARFuMS [18] (details in File S1). GenBank accession numbers for the full-length metagenomic fragments are KF626669 - KF630360.

Selected metagenomic fragments without known resistance function or with low identity to known resistance genes were validated by PCR-amplifying the open reading frame (ORF) in question from its library of origin (Table 2, Table S1 in File S1, details in File S1), ligated into pZE21, transformed into MegaX DH10B T1R, and selected on antibiotic media corresponding to its putative phenotype. ORFs were then PCR-amplified from colonies exhibiting a resistant phenotype and Sanger-sequenced to verify their identity. MICs for relevant antibiotics against clones with validated ORFs were determined via growth assays in MH broth [50].

### Statistical Analysis

Statistical analysis was performed using SAS software 9.1.3 [51]. Two-tailed Fisher’s Exact and Wilcoxon rank-sum Tests were used to test categorical and continuous data, respectively, for statistically significant differences between groups.

### Phylogenetic Analysis

Multiple alignment of predicted protein sequences >200aa in length was performed using MUSCLE (http://www.drive5.com/muscle/, March 2012) and approximate maximum-likelihood phylogenetic trees were built using FastTree (http://www.microbesonline.org/fasttree/, March 2012) and visualized with FigTree (http://tree.bio.ed.ac.uk/software/figtree/, March 2012). Beta-lactamases were assigned to an Ambler class and aminoglycoside resistance proteins were identified as aminotransferases, phosphotransferases, or bifunctional enzymes according to the best hit to the NCBI nr or Antibiotic Resistance Genes database (ARDB), and by PSI-BLAST (details in File S1).

## Supporting Information

Figure S1 **Number and Classification of Contigs with Known Resistance Genes.** The upper graph shows the number of contigs with a known resistance gene, separated by antibiotic selection, on a log scale. The lower graph shows the fraction of those contigs for each antibiotic selection condition that have a single resistance gene, a multidrug resistance element, and a resistance gene with a mobile element.
(TIF)

File S1 **Supporting Information.** Text Supplementary Materials and Methods in File S1: Supplementary Materials and Methods. Table S1 in File S1: Ages of fecal sample donors. Table S2 in File S1: PCR Primers used in this study. Each primer pair is assigned a number from 1–12 with the forward primer designated as “A” and the reverse primer designated as “B”. PCR templates and products are listed to the right of the primer sequences. Table S3 in File S1: Antibiotics used in functional metagenomic screens Antibiotic concentrations were selected according to the minimum observed inhibitory concentration (MIC) of MegaX DH10B T1R that had been transformed with unmodified pZE21. If the MIC for the MegaX DH10B T1R with pZE21 was lower than the sensitivity breakpoint for *Enterobacteriaceae* listed in the Clinical and Laboratory Standards Institute (CLSI) manual, the antibiotic concentration was set at the sensitivity breakpoint. The sole exceptions were ciprofloxacin, which was screened at the lowest observed MIC for MegaX DH10BT1R transformed with pZE21, which was below the breakpoint listed in the CLSI, and tigecycline, for which there are no standards in the CLSI manual for dilution susceptibility testing (disk diffusion only is described). A tigecycline concentration of 2 ug/mL was used to determine tigecycline nonsusceptibility in our *E. coli* host, as this far exceeds the reported range of susceptibility in wild-type *E. coli*. [53], [54] All concentrations are in ug/mL, and all antibiotics were added to Mueller-Hinton agar with 50 ug/mL of kanamycin (MH-Kan). Table S4 in File S1: Keywords used to categorize PARFuMS annotations. If any term in a given category (antibiotic resistance, transporter, or mobile element) appeared in the set of annotations for an ORF, the entire ORF was categorized accordingly. There was a hierarchy of categories so that the antibiotic resistance category superseded the transporter or mobile element categories (e.g. if one annotation was for an efflux pump, and another was for a multidrug-resistant efflux pump, the ORF would be classified as an antibiotic resistance gene). If an ORF was annotated with any of the terms in the column marked “flagged for further review”, the ORF was reviewed manually by performing a BLASTx with default settings against the NCBI nr database and against the Antibiotic Resistance Genes Database (ARDB) to determine if a resistance gene was present. Table S5 in File S1: Categories of metagenomic fragments based on keyword search of PARFuMS output. Each metagenomic fragment was assigned to one of the categories listed in the left column based on the assignment of its component ORFs, as listed in the left column. Contigs initially assigned as “unknown” were BLASTed against the NCBI nr/nt databases to identify any additional resistance genes; their ORFs were then re-categorized and the contigs reassigned accordingly. Table S6 in File S1: rRNA methylases identified in gentamicin screens. Contig names are listed as well as the functional annotation, the highest-scoring hit to the 16S rRNA methylase ORF in the NCBI nr database, as well as the identity to the top nr hit. The ORF from F19GE_3 was PCR-amplified and cloned into MegaX DH10B T1R E. coli, where it was found to confer extremely high-level resistance to amikacin and gentamicin. Table S7 in File S1: Selected chloramphenicol resistance genes. Contigs represented on this table were selected from the 78 total contigs with known resistance genes against chloramphenicol, and were included to illustrate the mechanistic diversity of chloramphenicol resistance genes in the infant and child age groups, and to provide interesting examples of synteny between chloramphenicol resistance genes and mobilization elements. The name of the contig, age group of the fecal donor, and annotation of the ORF according to the top hit against the NCBI nr database are included. Other ORFs of interest located on the same contig are found in the “Syntenic with” column. Table S8 in File S1: Selected tetracycline resistance genes. Contigs represented on this table were selected from the 110 total contigs with known resistance genes in this selection, and were included to illustrate the mechanistic diversity of tetracycline resistance genes in the infant and child age groups, and to provide interesting examples of synteny between tetracycline resistance genes and mobilization elements or other resistance genes. The name of the contig, age group of the fecal donor, and annotation of the ORF according to the top hit against the NCBI nr database are included. Other ORFs of interest located on the same contig are found in the “Syntenic with” column. Table S9 in File S1: Selected tigecycline resistance genes. Contigs on this table represent three of the seven contigs with known resistance genes in this selection, and were included to provide interesting examples of synteny between tigecycline resistance genes and mobilization elements or other resistance genes. The name of the contig, age group of the fecal donor, and annotation of the ORF according to the top hit against the NCBI nr database are included. Other ORFs of interest located on the same contig are found in the “Syntenic with” column. Table S10 in File S1: Selected trimethoprim resistance genes. Contigs represented on this table were selected from the 703 total contigs with known resistance genes in this selection, and were included to illustrate the mechanistic diversity of trimethoprim resistance genes in the infant and child age groups, and to provide interesting examples of synteny between trimethoprim resistance genes and mobilization elements or other resistance genes. The name of the contig, age group of the fecal donor, and annotation of the ORF according to the top hit against the NCBI nr database are included. Other ORFs of interest located on the same contig are found in the “Syntenic with” column. Table S11 in File S1: Selected trimethoprim-sulfamethoxazole resistance genes. Contigs represented on this table were selected from the 537 total contigs with known resistance genes in this selection, and were included to illustrate the mechanistic diversity of trimethoprim-sulfamethoxazole resistance genes in the infant and child age groups, and to provide interesting examples of synteny between trimethoprim-sulfamethoxazole resistance genes and mobilization elements or other resistance genes. The name of the contig, age group of the fecal donor, and annotation of the ORF according to the top hit against the NCBI nr database are included. Other ORFs of interest located on the same contig are found in the “Syntenic with” column. Table S12 in File S1: Selected cycloserine resistance genes. Contigs represented on this table were selected from the 956 total contigs with known resistance genes in this selection, and were included to illustrate the mechanistic diversity of cycloserine resistance genes in the infant and child age groups, and to provide interesting examples of synteny between cycloserine resistance genes and mobilization elements or other resistance genes. The name of the contig, age group of the fecal donor, and annotation of the ORF according to the top hit against the NCBI nr database are included. Other ORFs of interest located on the same contig are found in the “Syntenic with” column.
(PDF)
