# Supplementary figures and images for: Pediatric Fecal Microbiota Harbor Diverse and Novel Antibiotic Resistance Genes
Source: PLoS One. 2013 Nov 13;8(11):e78822. doi: 10.1371/journal.pone.0078822 (PMC3827270; doi:10.1371/journal.pone.0078822)

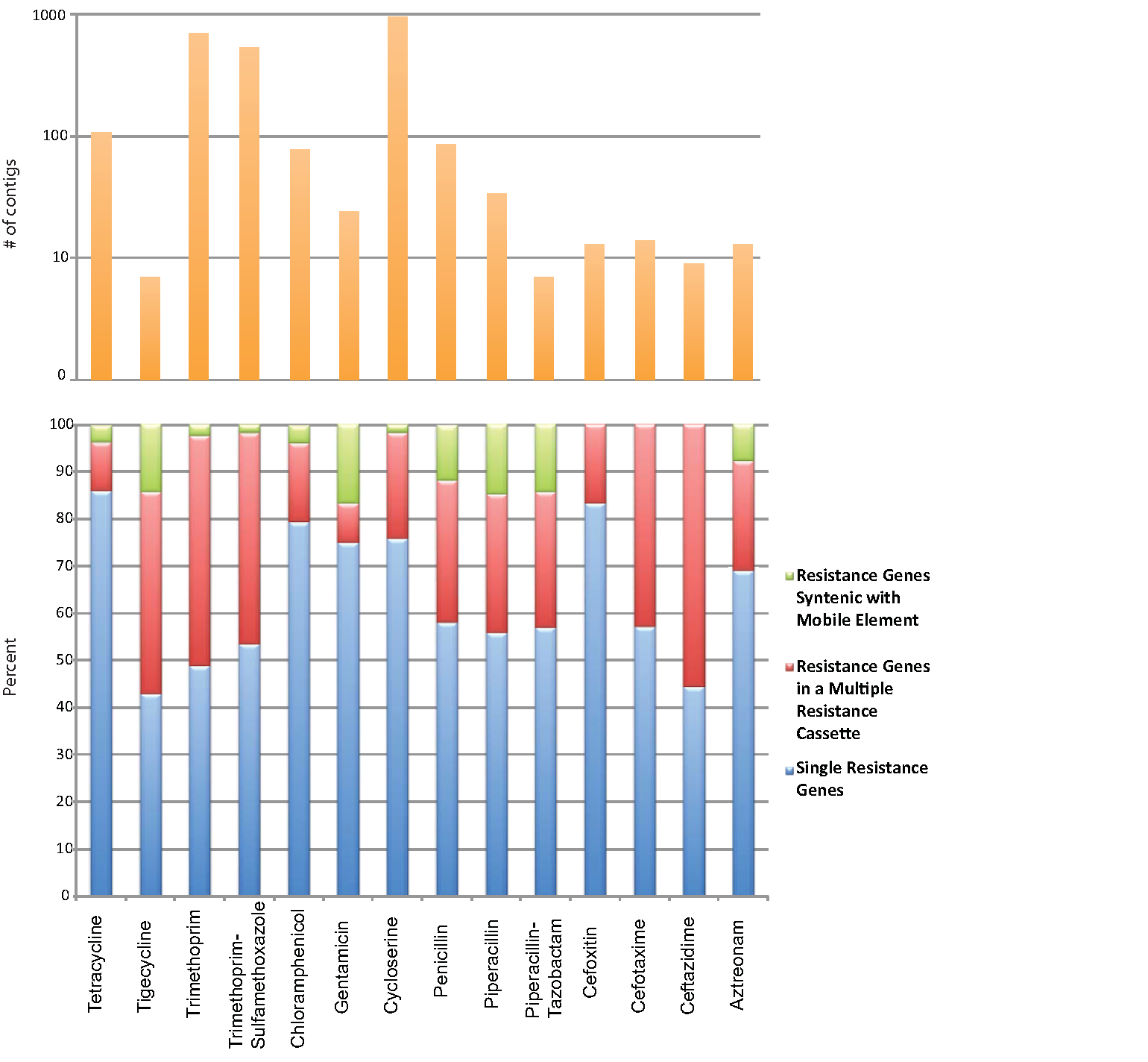

Supplement: Figure S1 — Number and Classification of Contigs with Known Resistance Genes. The upper graph shows the number of contigs with a known resistance gene, separated by antibiotic selection, on a log scale. The lower graph shows the fraction of those contigs for each antibiotic selection condition that have a single resistance gene, a multidrug resistance element, and a resistance gene with a mobile element. (TIF) [file pone.0078822.s001.tif]
