# Supplementary material for: Pediatric Fecal Microbiota Harbor Diverse and Novel Antibiotic Resistance Genes
Source: PLoS One. 2013 Nov 13;8(11):e78822. doi: 10.1371/journal.pone.0078822 (PMC3827270; doi:10.1371/journal.pone.0078822)
Supplement: File S1 — Supporting Information. Text Supplementary Materials and Methods in File S1: Supplementary Materials and Methods. Table S1 in File S1: Ages of fecal sample donors. Table S2 in File S1: PCR Primers used in this study. Each primer pair is assigned a number from 1–12 with the forward primer designated as “A” and the reverse primer designated as “B”. PCR templates and products are listed to the right of the primer sequences. Table S3 in File S1: Antibiotics used in functional metagenomic screens Antibiotic concentrations were selected according to the minimum observed inhibitory concentration (MIC) of MegaX DH10B T1R that had been transformed with unmodified pZE21. If the MIC for the MegaX DH10B T1R with pZE21 was lower than the sensitivity breakpoint for Enterobacteriaceae listed in the Clinical and Laboratory Standards Institute (CLSI) manual, the antibiotic concentration was set at the sensitivity breakpoint. The sole exceptions were ciprofloxacin, which was screened at the lowest observed MIC for MegaX DH10BT1R transformed with pZE21, which was below the breakpoint listed in the CLSI, and tigecycline, for which there are no standards in the CLSI manual for dilution susceptibility testing (disk diffusion only is described). A tigecycline concentration of 2 ug/mL was used to determine tigecycline nonsusceptibility in our E. coli host, as this far exceeds the reported range of susceptibility in wild-type E. coli. [53], [54] All concentrations are in ug/mL, and all antibiotics were added to Mueller-Hinton agar with 50 ug/mL of kanamycin (MH-Kan). Table S4 in File S1: Keywords used to categorize PARFuMS annotations. If any term in a given category (antibiotic resistance, transporter, or mobile element) appeared in the set of annotations for an ORF, the entire ORF was categorized accordingly. There was a hierarchy of categories so that the antibiotic resistance category superseded the transporter or mobile element categories (e.g. if one annotation was for an efflux pump, [file pone.0078822.s002.pdf]

**SUPPLEMENT**

**Table of Contents**


I. Materials and Methods: pp. 2-9

a. Construction and Functional Screening of Metagenomic Libraries: pp. 2-4
   i. Selection of fecal samples: p. 2
   ii. Metagenomic DNA extraction: p. 2
   iii. pZE21 Vector Preparation: pp. 2-3
   iv. Metagenomic Library Construction: pp. 3-4
   v. Functional Screening on Antibiotic Media: p. 4

b. Sequencing and Annotation: pp. 4-7
   i. Indexing of samples for Sequencing: pp. 4-6
   ii. Overview of the Parallel Annotation and Reassembly of Functional Metagenomic Selections (PARFuMS) computational pipeline: p. 6
   iii. Determining the reduction in sequencing cost afforded by PARFuMS: pp. 6-7

c. Data Analysis: p. 7
   i. Categorization of metagenomic fragments: p. 7
   ii. Creation of Beta-Lactamase and Aminoglycoside-modifying Enzyme Approximate Maximum-Likelihood Trees: p. 7

d. Experimental Validation of Novel Genes: pp. 7-9
   i. RBS-pZE21 Vector Preparation: p. 7
   ii. Cloning, Phenotypic Validation, and Sequence Confirmation of Novel Genes: pp. 7-8
   iii. Determination of MICs for Clones with Novel Resistance Genes: p. 9

II. Supplemental Tables: pp. 10-22

III. References: p. 23

# I. MATERIALS AND METHODS

## A. Construction and Functional Screening of Metagenomic Libraries

### *i. Selection of fecal samples*

22 fecal samples were selected from an archive of 455 pediatric fecal samples collected for a previous study. To ensure that a broad distribution of ages was represented and that the first year of live was particularly well-represented, one to two samples were chosen randomly within defined age categories (e.g. 0-3mo, 4-5years, >14 years). The age distribution of the 22 sample donors is listed in Table S1. Fecal DNA was extracted and metagenomic libraries were constructed and screened as previously described.(1, 2) The experimental protocols are briefly described below.

### *ii. Metagenomic DNA extraction*

Metagenomic DNA was isolated from 50-100mg of fecal material from each donor using 250uL of 0·1mm zirconium beads (BioSpec Products, cat#1107910), 210uL of 20% sodium-dodecyl-sulfate (SDS), 500uL of a custom buffer (100mM NaCl, 100mM Tris, 10mM EDTA), and 500uL phenol:chloroform:isoamyl alcohol (24:24:1; pH 7·9). Fecal cells were then lysed in this suspension using a Mini-Beadbeater (BioSpec Products) on the "homogenize" setting for a total of four minutes (bead-beating for two minutes, samples cooled on ice for two minutes, bead-beating for two minutes). The samples were then centrifuged at 13,000rpm and the aqueous phase was added to a phase-lock gel tube (5Prime, cat#230280) along with another 600uL of phenol:chloroform:isoamyl alcohol (24:24:1; pH 7·9). The aqueous phase was placed in an Eppendorf tube and the DNA was recovered from the solution using isopropanol precipitation. A final purification step using the QIAquick® PCR Purification kit (Qiagen) was performed using the manufacturer's protocols. DNA was quantitated using the NanoVue™ Spectrophotometer (Fisher Scientific).

### *iii. pZE21 Expression Vector Preparation*

The pZE21 expression vector(3) was linearized for library creation at the HincII cut site using PCR with Platinum® PFX DNA Polymerase (Invitrogen). The reaction mixture, which differed from the manufacturer's recommendations, is detailed below; primer sequences are listed in Table S2.

*Per 50uL PCR reaction*

- 10X PFX Amplification Buffer: 10uL
- 10mM dNTP Mix: 1·5uL
- 50mM Magnesium Sulfate: 1uL
- 10X $PCR_x$ Enhancer Solution: 5uL
- 10uM Forward primer (1A, Table S2): 0·75uL
- 10uM Reverse primer (1B, Table S2): 0·75uL
- 100pg/uL pZE21: 1uL
- Nuclease-free water: 29·6uL
- Platinum® PFX DNA Polymerase 2·5U/uL: 0·4uL

*PCR cycling conditions*

- 95ºC for 10 minutes
- 95ºC for 45 seconds
- 55ºC for 45 seconds
- 72ºC for 2·5 minutes
- Go to second step; repeat 32 times
- 72ºC for five minutes
- 4ºC indefinitely

The PCR product was subjected to gel electrophoresis (1% agarose, 0·5x Tris-Borate-EDTA (TBE), GelGreen™ (Biotium, Inc.)) and the 2kb product was excised from the gel. Gel purification was performed using the QIAquick® Gel Extraction kit (Qiagen) following their recommended protocols. The purified product was then treated with calf intestinal alkaline phosphatase (CIP, New England BioLabs Inc.). The reaction mixture, which differed from the manufacturer's standard protocol, is listed below.

*Per 50uL reaction*

- CIP (10U/uL): 5uL
- 10x NEBuffer 3 (New England Biolabs, Inc.): 5uL
- Gel-purified pZE21: 30 uL
- Nuclease-free water: 10uL

The reaction was incubated overnight at 37ºC, then at 70ºC for 15 minutes. The product was then purified with the QIAquick® PCR Purification kit (Qiagen) using the manufacturer's protocols and quantitated with the NanoVue™ Spectrophotometer (Fisher Scientific).

***iv. Metagenomic Library Construction***

Metagenomic DNA was diluted in Buffer EB (Qiagen) to a concentration of 5ug/100uL and sheared using the Bioruptor® XL (Diagenode). For each sample, 20ug of metagenomic DNA was aliquoted into four 1·6-mL tubes and sheared on low power for a total of 29 seconds (five cycles of 5.8 seconds on and 18.2 seconds off). The sheared DNA was then size-selected via gel electrophoresis (1% agarose, 0·5x Tris-Borate-EDTA (TBE), GelGreen™ (Biotium, Inc.)) at 70V for 130 minutes. A gel slice covering the 2-5kb size range was removed from the gel and metagenomic DNA was recovered from the slice using the QIAquick® Gel Extraction kit (Qiagen) and following the manufacturer's recommended protocol. The size-selected metagenomic DNA was then end-repaired using the END-IT™ DNA End Repair kit (Epicentre®) using the manufacturer's recommended protocol. The end-repaired metagenomic DNA was then purified using the QIAquick® PCR Purification kit (Qiagen) following the manufacturer's protocol, eluted in sterile nuclease-free water, and concentrated to a volume of ~10uL using the SpeedVac® (ThermoScientific). The concentrated metagenomic DNA was quantitated using the Qubit® dsDNA HS Assay Kit (Invitrogen) and the Qubit® 2·0 Fluorometer (Invitrogen).

Blunt ligation of the metagenomic DNA and linearized pZE21 was performed with the FAST-LINK™ DNA Ligation kit (Epicentre®) following the manufacturer's recommended protocol for blunt ligation. A 1:5 molar ratio of pZE21 to metagenomic DNA was maintained in all reactions. Ligated plasmids were desalted using a nitrocellulose membrane (Millipore) and the entire reaction was added to 25uL of MegaXDH10B™ T1R™ Electrocompetent Cells (Invitrogen). Electroporation was performed using standard procedure for a 1mm cuvette, and the new metagenomic library was shaken at 37ºC for 1 hour before a 2uL aliquot was removed and used to plate titers on Luria-Bertani agar with 50ug/mL of kanamycin (the pZE21 expression vector has a kanamycin resistance cassette). The remainder of the library was transferred to 50mL of LB broth with 50ug/mL kanamycin (LB-Kan) and amplified at 30°C until the solution reached an optical density of 0·7-0·9 (typically 16-20 hours). The amplified library was gently centrifuged (3000rpm for 8 minutes), reconstituted in LB-Kan with 15% glycerol, and stored at -80ºC. Amplified library titers were determined by plating on LB-Kan agar prior to freezing.

Colony PCR was done using the titer plates obtained immediately after electroporation. 12 colonies were picked at random from the titer plates and the cells were lysed in 50uL nuclease-free water. For each colony, the following PCR reaction was performed using the *Taq* DNA polymerase with Thermopol kit (New England Biolabs, Inc).

*Per 25uL PCR reaction*

- PCR template (lysed colony in nuclease-free water): 2·5uL
- 10x Thermopol Buffer: 2·5uL
- 10mM dNTP mix: 1uL
- Forward Primer (3A, Table S2): 1uL
- Reverse Primer (3B, Table S2): 1uL
- Nuclease-free water: 16·5 uL
- Taq polymerase 5U/uL: 0·5uL

*PCR cycling conditions*

- 94ºC for 10 minutes
- 94ºC for 45 seconds
- 55ºC for 45 seconds

- 72ºC for 5 minutes 30 seconds
- Go to second step; repeat 24 times
- 72ºC for 5 minutes
- 4ºC indefinitely

The colony PCR product was visualized on a 1% agarose gel containing ethidium bromide. If the pZE21 had self-ligated with no insert, a 300bp band was seen; otherwise the bands seen reflected the size of the metagenomic fragment taken up by that clone + 300bp. The fraction of colony PCR reactions with bands >800bp in size seen on the gel, reflecting the approximate fraction of metagenomic fragments >500bp in size present in the library, was used to generate a correction factor used in the estimation of library size (e.g. if nine of the twelve clones had bands >800bp in size, the resultant correction factor would be 0·75). The size of each library, in gigabases (GB) was estimated using the following formula:

Library Size (GB) = Total Clones * correction factor * approximate insert size

(Total clones = cfu/mL of the library 1 hour after electroporation, as determined from the titer plates. Correction factor is determined by visualization of colony PCR product, and the average insert size is estimated at 2000bp.

***v. Functional Screening on Antibiotic Media***

Prior to plating on antibiotic-containing growth media, the concentration of the metagenomic libraries was adjusted so that each 100uL of plating solution contained 10x the number of total unique clones estimated in the library at 1 hour after electroporation (for a library with 1 x $10^6$ clones following electroporation, the stock made for plating would contain 1 x $10^7$ cfu/100uL, or 1 X $10^8$ cfu/mL). If the concentration of the frozen library stock was higher than the desired concentration, the library was diluted with LB-Kan; if it was lower than the desired concentration, the cells were pelleted by gentle centrifugation at 3000rpm and reconstituted in an appropriate volume of LB-Kan. Libraries were plated on Mueller-Hinton agar with 50ug/mL of kanamycin (MH-Kan) and additional antibiotics at concentrations listed in Table S3. One plate was used for the selection of each library for resistance against a particular antibiotic. A control sample of the MegaX DH10B T1R strain with unmodified pZE21 was plated on each antibiotic at the time of screening to ensure the concentration of antibiotic used entirely inhibited the growth of clones with only pZE21. Plates were incubated for 24 hours at 37°C before counting the resistant colonies. Trimethoprim and Trimethoprim-sulfamethoxazole plates and controls were incubated for 48 hours because resistant clones were too small to count accurately at 24 hours. After the colonies were counted, 1.5mL of LB-Kan with 15% glycerol was added in two 750µl aliquots to the plates and the colonies were gently removed from the agar with a sterile L-shaped cell-spreader. The slurry of functionally selected clones was then removed from the surface of the plate by pipette aspiration and was stored at -80°C.

**B. Sequencing and annotation**

Functionally selected metagenomic fragments were barcoded for sequencing, then reassembled and annotated with the PARFuMS computational pipeline. The protocol is briefly outlined below.

***i. Indexing samples for sequencing***

DNA was extracted from 300uL of functionally selected clones by pelleting the cells with high-speed centrifugation (13,000 rpm), removing the supernatant with a pipet, rinsing the pellet with 1mL of nuclease-free water, and then lysing the cells by resuspending them in 30uL of nuclease-free water and freezing at -20ºC. The cellular debris was then pelleted by high-speed centrifugation and the supernatant was used as a template for PCR amplification of functionally-selected metagenomic fragments. For each selection condition (one subject vs. one antibiotic comprises a single selection condition), the following PCR reaction was set up:

*Per 25uL reaction*

- PCR template (lysed colony in nuclease-free water): 3uL
- 10x Thermopol Buffer: 2·5uL
- 10mM dNTP mix: 0·5uL
- Forward Primer (4A, Table S2): 1·5uL
- Reverse Primer (4B, Table S2): 1·5uL

- Nuclease-free water: 15·5 uL
- Taq polymerase 5U/uL: 0·5uL

*PCR cycling conditions*
- 94ºC for 10 minutes
- 94ºC for 45 seconds
- 55ºC for 45 seconds
- 72ºC for 5 minutes 30 seconds
- Go to second step; repeat 24 times
- 72ºC for 5 minutes
- 4ºC indefinitely

PCR cycling conditions were identical to the colony PCR protocol described above. The PCR product was purified using the QIAquick® PCR Purification kit (Qiagen) using the manufacturer's protocols and quantitated with the NanoVue™ Spectrophotometer (Fisher Scientific). The DNA was then diluted to a concentration of 50ng/uL and 100uL of each sample was placed into a 600uL Eppendorf tube. These 100uL aliquots of DNA were sheared to ~160bp fragments using the Bioruptor® XL (Diagenode) on high power for a total of 45 minutes (90 cycles of 30 seconds on and 30 seconds off). The sheared DNA was then concentrated using the QIAquick® PCR Purification kit (Qiagen) with MinElute columns. After measuring the concentration with the NanoVue™ Spectrophotometer (Fisher Scientific), the sheared DNA was diluted to a concentration of 40ng/uL and the ends were blunted using the following reaction:

- Sheared DNA (40ng/uL): 10uL
- 10X T4 DNA ligase buffer with 10mM ATP (New England Biolabs, Inc.): 2uL
- T4 Polynucleotide Kinase 10U/uL (New England Biolabs, Inc.): 0·5uL
- Klenow DNA polymerase 5U/uL (New England Biolabs, Inc.): 0·5uL
- 10mM dNTP mix: 0·1uL
- Nuclease-free water: 6·9uL

After the end-repair reaction had been incubated at 25ºC for 30 minutes then at 75ºC for 20 minutes, 1uL of shrimp alkaline phosphatase 1U/uL (Promega) was added and the reaction incubated at 37ºC for 30 minutes then at 75ºC for 30 minutes to dephosphorylate any remaining dNTPs. Each blunted DNA fragment then had an adenosine added to the 3' end by adding 6·4uL of 1X T4 DNA ligase buffer with 10mM ATP (diluted from 10X stock; New England Biolabs, Inc.), 0.6uL of dATP 5mM (Promega), and 2uL of Klenow fragment (3' → 5' exo-) 5U/uL (New England Biolabs, Inc.): 2uL and incubating at 37ºC for 30 minutes then at 75ºC for 20 minutes. In a separate tube, 1·4uL of a 25uM solution comprised of (i) a universal forward adapter and (ii) reverse adapters indexed with unique DNA tags specific to a given selection (adapters mixed in equal proportions) was added to 5uL 10X T4 DNA ligase buffer with 10mM ATP (New England Biolabs, Inc.) and 12uL of nuclease-free water and heated to 94ºC for 2 minutes and slowly cooled to room temperature to anneal the adapters. The annealed adapters were then mixed with 1uL of T4 DNA ligase 2,000U/uL (New England Biolabs, Inc.), and added to the end-repaired DNA solution (a unique indexed adapter was assigned to DNA fragments from each selection condition). The adapter ligation reaction was incubated for 60 minutes at 16ºC, then for 10 minutes at 65ºC, and then the indexed fragments were purified using the QIAquick® PCR Purification kit (Qiagen) with MinElute columns.

Size selection of the barcoded DNA fragments was performed by loading the samples pooled in groups of 4 on a gel (2% agarose, 1x TBE, GelGreen™ (Biotium, Inc.) and electrophoresing at 120V for 2 hours. The size range that corresponded to sheared metagenomic DNA fragments of length ~160bp in length was excised and the DNA was recovered using the QIAquick® Gel Extraction kit (Qiagen) with the MinElute columns. Size-selected indexed DNA fragments were PCR-enriched using the following reaction:

*Per 25uL reaction*
- 2x Phusion HF MasterMix (Finnzymes): 12·5uL
- Illumina PCR Primer Mix 10uM: 1uL
- Nuclease-free water: 10·5uL

- Size-selected barcoded DNA: 1uL

*PCR cycling conditions*

- 98ºC for 30 seconds
- 98ºC for 10 seconds
- 65ºC for 30 seconds
- 72ºC for 30 seconds
- Go to second step; repeat 17 times
- 72ºC for 5 minutes
- 4ºC indefinitely

The PCR product was then size-selected on a 2% agarose gel 1X TBE gel with GelGreen (Biotium) to remove primer-dimers. DNA was recovered from the gel slices using the QIAquick® PCR Purification kit (Qiagen) with the MinElute columns, and the concentration of the pooled, size-selected, barcoded DNA was measured using the Qubit® dsDNA HS Assay Kit (Invitrogen) and the Qubit® 2·0 Fluorometer (Invitrogen). 10 nanomoles of each PCR product were then aliquoted into a pooled sample that was submitted to the Genome Technology Access Center (GTAC, Washington University in St Louis) for Illumina Hi-Seq Paired-End 101bp sequencing.

***ii. Overview of the Parallel Annotation and Reassembly of Functional Metagenomic Selections (PARFuMS) computational pipeline***

To simultaneously assemble metagenomic fragments from multiple selections in parallel, we implemented an iterative assembly approach. Intermediate-length contigs were generated using multiple rounds of assembly with the short-read assemble Velvet,(4) and then further assembled into full-length contigs using the long-read assembler Phrap.(5) Assembly with Velvet cycles through three iterations: in the first iteration, assembly occurs using all reads while in the second and third, only reads that were not present in any previously assembled contig are used. Each round of assembly is divided into jobs of a defined number of reads, with each set of reads assembled in parallel, and all assembled contigs joined at the end of each round. As PARFuMS progresses through assemblies, the number of reads per job changes, decreasing in later assemblies as dataset complexity is reduced. This approach regulates the coverage across contigs, ensuring the number of reads with sequencing error in any given assembly remains low, avoiding the inappropriate division of contigs. Following each iteration of assembly, redundant contigs are collapsed to one sequence and chimeric assemblies split using a window-based coverage approach. This contig set represents the final Velvet-assembled contigs, which are then passed to Phrap for two iterations of further assembly. The first iteration assembles raw Velvet output into more complete contigs which are subsequently linked together using raw reads that bridge the ends of two sequences. The final assembly of Phrap uses the linked contigs as input, outputting contigs that are subsequently annotated via similarity to the COG functional database.(6) Finally, contigs are joined based on both sequence similarity and common annotation to generate the final sequence set, which is re-annotated through BlastX-similarity to the COG functional database. PARFuMS performance has been benchmarked on previously Sanger-sequenced human fecal metagenomic libraries,(2) and we observe not only recapitulation of the Sanger-sequenced results, but also importantly up to 20% more novel sequence recovery from libraries of similar size to ones screened in this paper.(7)

***iii. Determining the reduction in sequencing cost afforded by PARFuMS***

A single lane of sequence data from the Illumina Hi-Seq was estimated to cost $3250 (current price at GTAC). Assuming PARFuMS requires 700,000 reads per selection (in reality, PARFuMS requires fewer reads) and 140 million sequencing reads are generated per lane, this scheme can process 200 selections for $3250. To calculate how many selections could be processed for the same price using traditional Sanger-based sequencing methods, several assumptions regarding reagent-cost were made. First, it was assumed that six Sanger sequencing reactions would be needed to entirely sequence any given metagenomic insert from a resistant clone (four custom primers and two universal primers complementary to the ends of the expression vector). At an average cost of $3 per sequencing reaction (current price at Beckman Coulter) and $2 per custom primer (current price at Integrated DNA Technologies), sequencing the metagenomic insert from any given clone would cost $26. If the average selection yields 100 resistant clones (an

underestimate compared to the mean colony counts observed in most antibiotic selections), sequencing a single experiment would cost $2600. Therefore, 1.25 selections could be sequenced for $3250, for which price 200 functional selections can be processed with PARFuMS, making the per-selection cost of PARFuMS <1% of the cost incurred using Sanger-based methods.

**C. Data Analysis**
The PARFuMS output was parsed as described below.

***i. Categorization of metagenomic fragments***
The PARFuMS output, which contains a set of functional annotations from the COG database for each ORF on every assembled contig, was queried for a set of keywords (Table S4) and each ORF was categorized as either a known antibiotic resistance gene, a gene encoding a transporter/efflux protein, a mobile genetic element, or “other” (the category “other” includes all annotations besides the three listed). Based on the assignment of the ORFs on a given contig, each contig was classified (Table S5) as either bearing a single resistance gene, bearing a multidrug resistance element (defined as either bearing two unique resistance genes or a resistance gene and a transporter, to best highlight contigs encoding multiple resistance mechanisms), bearing a resistance gene with a mobile element, bearing a transporter/efflux protein only, or unknown (not bearing any ORFs assigned as a resistance gene, transporter, or mobile element). The total number of contigs bearing a known resistance gene, sorted by antibiotic, and the associated proportions of contigs with a single resistance gene, contigs with multidrug resistance elements, and contigs with resistance genes associated with mobile elements are depicted in Figure S1.

***ii. Creation of Beta-Lactamase and Aminoglycoside-modifying Enzyme Approximate Maximum-Likelihood Trees***
Two datasets comprised of the predicted amino acid sequences associated with all genes identified that encoded (1) beta-lactamases or (2) aminoglycoside acetyltransferases and phosphotransferases were selected for analysis. Partially-assembled sequences <200aa in length were excluded from these datasets. Each dataset was BLASTed against itself and any duplicate genes (100% amino acid identity with equal sequence lengths) were collapsed to one sequence. All remaining sequences were classified (Ambler classification for beta-lactamases; acetyltransferase, phosphotransferases, or bifunctional for aminoglycoside-modifying enzymes) using the best hit to the NCBI nr database, or the Antibiotic Resistance Genes Database (ARDB). PSI-BLAST was also performed on all aminoglycoside-modifying enzymes and on all beta-lactamases with less than 90% identity to any known beta-lactamase, to confirm categorizations. Multiple alignments on the two datasets were done using Muscle v3.7.(8) Subsequently, FastTree v.2.1(9) was used to create an approximate maximum-likelihood tree. Default parameters were used for both Muscle v3.7 and FastTree v2.1. Regions of the trees (Figs. 3, 4) corresponding to Ambler classes (beta-lactamases) or predicted enzyme activity (aminoglycoside-modifying enzymes) were overlaid with color.

**D. Experimental Validation of novel genes**
Selected genes with putatively novel resistance functions were amplified from the metagenomic libraries and then subjected to phenotypic assays to validate their function and Sanger–sequenced to confirm their identities.

***i. RBS-pZE21 Vector Preparation***
The HincII cut site is not appropriately spaced relative to the pZE21 ribosomal binding site to allow for optimal transcription; accordingly RBS-pZE21, a linearized pZE21 derivative with optimized spacing between the insertion site and the ribosomal binding site was created for the purpose of cloning individual ORFs. RBS-pZE21 was created using a different set of primer pairs than the pZE21 used for library creation (primers 2A/2B, Table S2); preparation of RBS-pZE21 was otherwise exactly as described for the standard pZE21 used for library creation.

***ii. Cloning, Phenotypic Validation, and Sequence Confirmation of Novel Genes***
Four genes were selected for phenotypic validation: two MFS transporters found in tetracycline selections with >98% identity to a *Bifidobacterium* MFS transporter not known to confer tetracycline resistance, a 16S rRNA methylase with only 42% identity to any known rRNA methylase, and a NUDIX hydrolase,

which acts early in the pterin branch of the folate synthesis pathway, in a trimethoprim-sulfamethoxazole selection. Functionally selected clones that had been scraped from each of the selection conditions (named 21TE, 30TE, 19GE, and 30TRSX, respectively) and frozen, were lysed as described in the section labeled "Indexing samples for sequencing" and the metagenomic fragments were PCR-amplified as follows.

*Per 25uL PCR reaction*

- PCR template (lysed slurry of functionally selected clones): 2·5uL
- 10x Thermopol Buffer: 2·5uL
- 10mM dNTP mix: 1uL
- Forward Primer (4A, Table S2): 1uL
- Reverse Primer (4B, Table S2): 1uL
- Nuclease-free water: 16·5 uL
- Taq polymerase 5U/uL: 0·5uL

*PCR cycling conditions*

- 94ºC for 10 minutes
- 94ºC for 45 seconds
- 55ºC for 45 seconds
- 72ºC for 5 minutes 30 seconds
- Go to second step; repeat 24 times
- 72ºC for 5 minutes
- 4ºC indefinitely

The PCR product was purified with the QIAquick® PCR Purification kit (Qiagen) and 1uL of the PCR product was used as template for another PCR reaction using primers specific to the full-length contigs bearing the four genes of interest. As shown in Table S3, primer pair 5A/5B was used to amplify the 19Gent contig from the 19GE PCR product; primer pair 6A/6B was used to amplify the 21MFSBif contig from the 21TE PCR product; primer pair 7A/7B was used to amplify the 30MFSBif contig from the 30TE selection; and primer pair 8A/8B was used to amplify the 30Nudix contig from the 30TRSX PCR product. Each of these PCR reactions was done using the same reaction conditions as described above. The PCR product was purified with the QIAquick® PCR Purification kit (Qiagen) and visualized on a 1% agarose gel stained with ethidium bromide (EB) to verify that the product was of the correct size. The product was then used as a PCR template for amplification of the ORFs. PCR conditions were the same as for the full-length fragments, except the extension time was shortened to 2 minutes. As shown in Table S3, primer pair 9A/9B was used to amplify 19GentORF from the 19Gent contig; primer pair 10A/10B was used to amplify 21MFSORF from the 21MFSBif contig; primer pair 11A/11B was used to amplify 30MFSORF contig from the 30MFSBif contig; and primer pair 12A/12B was used to amplify 30NudixORF from the 30Nudix contig. The ORF PCR product was then purified with the QIAquick® PCR Purification kit (Qiagen) and visualized on a 1% agarose gel with ethidium bromide with EB. ORF PCR products verified to be the correct size were then ligated into RBS-pZE21 using the FAST-LINK™ DNA Ligation kit (Epicentre®) exactly as described for metagenomic library preparation. Dialysis of the ligation reactions and electroporation were also done precisely as described earlier. Following electroporation, the transformants were allowed to recover at 37ºC in a shaking warmer for 1 hour, then they were amplified overnight in 50mL Mueller-Hinton broth with 50ug/mL of kanamycin and the antibiotic appropriate to the predicted ORF function at a concentration listed in Table S3 (gentamicin for 19GentORF, tetracycline for 21MFSORF and 30MFSORF, and trimethoprim-sulfamethoxazole for 30NudixORF). A 100uL aliquot of each sample was plated on Mueller-Hinton agar with 50ug/mL of kanamycin and the same ORF-specific antibiotic that the sample had been amplified in overnight. The remainder of the amplified stock was pelleted and reconstituted in LB-Kan with 15% glycerol and stored at -80ºC. Controls (MegaX DH10B T1R with unmodified pZE21) were plated at the same time on separate plates with equivalent antibiotic concentrations. After 24 hours (48 for 30Nudix), several colonies were picked off of each plate and the metagenomic fragment was amplified using the colony PCR protocol described in the section on preparing metagenomic libraries. The PCR product was purified using the QIAquick® PCR Purification kit (Qiagen) and visualized on a 1% agarose EB gel to confirm that the fragment was the correct size. Fragments were

then sent for Sanger sequencing using the colony PCR primers (primer pair 3A/3B) and the identities of the functionally validated clones were identified.

***iii. Determination of MICs for Clones with Novel Resistance Genes(10)***

Freezer stocks from the four clones (19GentORF, 21MFSORF, 30MFSORF, and 30Nudix) which were functionally validated, and whose identities were verified using Sanger sequencing, were inoculated into Mueller-Hinton broth with 50ng/uL of kanamycin (MH-Kan) for MIC testing in 96-well culture plates. In all cases, a control organism (MegaX DH10B T1R with unmodified pZE21) was subjected to identical culturing conditions. Culture plates were incubated at 37C for 48 hours and growth rates were observed during the entire period. 19GentORF was tested in MH-Kan with gentamicin (concentration range 16 – 1024 ug/mL), amikacin (concentration range 32 – 2048ug/mL), and spectinomycin (concentration range 2-128 ug/mL). The *E. coli* transformant with 19GentORF grew in all concentrations of amikacin and gentamicin tested (the control was inhibited at 16ug/mL for gentamicin and 32ug/mL amikacin). Both 19GentORF and the control were inhibited at 32ug/mL of spectinomycin. The two MFS transporters, 21MFSORF and 30MFSORF, were tested in MH-Kan with tetracycline (concentration range 4 – 256 ug/mL), oxytetracycline (concentration range 2 – 128 ug/mL), and tigecycline (concentration range 0·5 - 32ug/mL). Both 21MFSORF and 30MFSORf had MICs of 32ug/mL in tetracycline (control inhibited at 4ug/mL) and 0·5ug/mL in tigecycline (control also inhibited at 0·5ug/mL). 21MFSORF had an MIC of 64ug/mL in oxytetracycline, while 30MFSORF had an MIC of 32ug/mL in oxytetracycline (control inhibited at 2 ug/mL). 30Nudix was tested in MH-Kan with trimethoprim (concentration range 16-1024 ug/mL) and trimethoprim-sulfamethoxazole (concentration range 2/38 – 128/2432 ug/mL). The MIC for trimethoprim was 512 ug/mL (control inhibited at 16ug/mL) and 32/604 for trimethoprim-sulfamethoxazole (control inhibited at 2/38 ug/mL).

## II. SUPPLEMENTAL TABLES

## Table S1: Ages of Fecal Sample Donors

| Sample ID | Age (years) |
|---|---|
| F15 | 0·09 |
| F29 | 0·17 |
| F32 | 0·36 |
| F07 | 0·48 |
| F34 | 0·56 |
| F09 | 0·75 |
| F30 | 0·90 |
| F05 | 1·02 |
| F16 | 2·18 |
| F06 | 2·56 |
| F24 | 4·29 |
| F21 | 4·58 |
| F22 | 5·36 |
| F33 | 5·92 |
| F26 | 6·39 |
| F28 | 7·02 |
| F19 | 8·70 |
| F04 | 8·99 |
| F11 | 10·71 |
| F23 | 11·00 |
| F12 | 15·05 |
| F18 | 19·62 |

## Table S2: PCR Primers Used in this Study

| Primer | Primer Name | Primer Sequence | PCR Template | PCR product |
|---|---|---|---|---|
| 1A | pZE21_126_146FOR | 5'- GAC GGT ATC GAT AAG CTT GAT | pZE21 | Linearized pZE21 |
| 1B | pZE21_111_125rcREV | 5'- GAC CTC GAG GGG GGG | | |
| 2A | pZE21forORFs_FOR | 5'- TAG AGG CAT CAA ATA AAA CGA AAG G | Linearized pZE21 | RBS-pZE21 suitable for cloning in single ORFs |
| 2B | pZE21forORFs_rcREV | 5'- GGT ACC TTT CTC CTC TTT AAT GAA T | | |
| 3A | pZE21_49_70_FOR | 5'- GAT ACT GAG CAC ATC AGC AGG A | Lysed clones from a metagenomic library | Metagenomic fragments (used for determining library size) |
| 3B | pZE21_348_369_rcREV | 5'- CCT GAT TCT GTG GAT AAC CGT A | | |
| 4A | pZE21_111_125_FOR | 5'- CCC CCC CTC GAG GTC | Lysed clones or plasmids isolated from a functionally selected metagenomic library | Functionally selected metagenomic fragments |
| 4B | pZE21_126_146_rcREV | 5'- ATC AAG CTT ATC GAT ACC GTC | | |
| 5A | 19Gent_FOR | 5'- AGT ATT CGC CCT TTT CCA CA | Functionally selected metagenomic fragments (19GE) | Full-length contig bearing novel 16S rRNA methylase (19Gent) |
| 5B | 19Gent_rcREV | 5'- ACC CTC GAG GTC CCA GAT AA | | |
| 6A | 21MFSBif_FOR | 5'- TGC GCA AAT CTG GTA GTT GA | Functionally selected metagenomic fragments (21TE) | Full-length contig bearing *Bifidobacterium*-identical MFS transporter (1) (21MFSBif) |
| 6B | 21MFSBif_rcREV | 5'- GCT CCC GTC TGA ATG GAA TA | | |
| 7A | 30MFSBif_FOR | 5'- CAT CCA TGA CCG CCT CG | Functionally selected metagenomic fragments (30TE) | Full-length contig bearing *Bifidobacterium*-identical MFS transporter (2) (30MFSBif) |
| 7B | 30MFSBif_rcREV | 5'- ATC TCA TCG ATT TCG GCA TC | | |
| 8A | 30Nudix_FOR | 5'- GGC ACA CTT GGC ATC CTT AC | Functionally selected metagenomic fragments (30TRSX) | Full-length contig bearing *Lachnospiraceae*-identical NUDIX hydrolase (30Nudix) |
| 8B | 30Nudix_rcREV | 5'- CGG CCT ACA TCA CCA AAA TC | | |
| 9A | 19GentORF_FOR | 5'- ATG GAG TTT TTG CGG AAA TGA G | Full-length contig bearing novel 16S rRNA methylase (19Gent) | Novel 16S rRNA methylase ORF (19GentORF) |
| 9B | 19GentORF_rcREV | 5'- GCC GGG GCA TGT TCA A | | |
| 10A | 21MFSORF_FOR | 5'- ATG TCT TCT AGT AGT ACA ACA ACT G | Full-length contig bearing *Bifidobacterium*-identical MFS transporter (1) (21MFSBif) | *Bifidobacterium*-identical MFS transporter (1) ORF (21MFSORF) |
| 10B | 21MFSORF_rcREV | 5'- TCA GGC CGC TGT TCG | | |
| 11A | 30TE_MFSORF_FOR | 5'- ATG GAG AAC ACT CGT ATG TCT T | Full-length contig bearing *Bifidobacterium*-identical MFS transporter (2) (30MFSBif) | *Bifidobacterium*-identical MFS transporter (2) ORF (30MFSORF) |
| 11B | 30TE_MFSORFrcREV | 5'- TCA GGC CGC TGT TCG | | |
| 12A | 30Nudix_ORF_FOR | 5'- TGA CCT ACC GGA GGA GGA | Full-length contig bearing *Lachnospiraceae*-identical NUDIX hydrolase (30Nudix) | *Lachnospiraceae*-identical NUDIX hydrolase ORF (30NudixORF) |
| 12B | 30Nudix_ORF_rcREV | 5'- TTA TGC GTT CCG CAG CA | | |

## Table S3: Antibiotics used in functional metagenomic screens

| Antibiotic | Class | Concentration (ug/mL) |
|---|---|---|
| Gentamicin | Aminoglycoside | 16 |
| Penicillin | Beta-lactam | 128 |
| Piperacillin | Beta-lactam | 16 |
| Piperacillin-Tazobactam | Beta-lactam with beta-lactamase inhibitor | 16/4 |
| Cefoxitin | Beta-lactam (cephamycin; activity like 2nd generation cephalosporin) | 32 |
| Cefotaxime | Beta-lactam (3rd generation cephalosporin) | 8 |
| Ceftazidime | Beta-lactam (3rd generation cephalosporin antipseudomonal) | 16 |
| Cefepime | Beta-lactam (4th generation cephalosporin) | 8 |
| Aztreonam | Beta-lactam (monobactam) | 8 |
| Meropenem | Beta-lactam (carbepenem) | 16 |
| Tetracycline | Tetracycline | 8 |
| Tigecycline | Tetracycline derivative (glycylcycline) | 2 |
| Chloramphenicol | Amphenicol | 8 |
| D-Cycloserine | D-alanine analog | 32 |
| Colistin | Polymyxin | 8 |
| Trimethoprim | Folate synthesis inhibitor | 8 |
| Trimethoprim-sulfamethoxazole | Folate synthesis inhibitor | 2/38 |
| Ciprofloxacin | Quinolone | 0·5 |

## Table S4: Keywords used to categorize PARFuMS annotations

| Antibiotic Resistance | Transporter | Mobile Element | Flagged for further review |
|---|---|---|---|
| beta-lactamase | Efflux | transposase | RNA methylase |
| beta lactamase | Transporter | transposon | RNA methyltransferases |
| Penicillin | Transport | conjugative | Fumarate reductases |
| Methicillin | Pump | Integrase | Monooxygenase |
| Cephalosporinase | Permease | Integron | SoxS |
| Vancomycin | Antiporter | recombinase | Right origin |
| Aminoglycoside | Symporter | Conjugal | |
| Gentamicin | | mobilization | |
| Kanamycin | | recombination | |
| Tobramycin | | Plasmid | |
| Streptomycin | | | |
| Chloramphenicol | | | |
| tetracycline resistance | | | |
| Multidrug | | | |
| dihydrofolate reductases | | | |
| thymidylate synthase | | | |
| thymidylate synthetase | | | |
| sul1 | | | |
| sul2 | | | |
| sul3 | | | |
| dihydropteroate synthase | | | |
| Macrolide | | | |
| Antibiotic | | | |
| Drug | | | |
| Clindamycin | | | |
| Bleomycin | | | |
| Cycloserine | | | |
| D-alanine | | | |

## Table S5: Classification of metagenomic fragments based on keyword search of PARFuMS output

| **Metagenomic Fragment Category** | **Component ORF Classification** |
|---|---|
| Single Resistance Gene | One known resistance gene |
| Multidrug Resistance Elements | >1 known resistance gene OR 1 known resistance gene and >=1 efflux pump |
| Resistance Gene with Mobile Element | >=1 known resistance gene and >=1 mobile element |
| Transporter only | >=1 known transporter or efflux pump. No known resistance genes. |
| Unknown | No known resistance genes or transporters. |

# Table S6: rRNA methylases identified in gentamicin selections

| Contig Name | Annotation | Top hit (nr) | % ID | % cov |
|---|---|---|---|---|
| F04GE_5 | 16S rRNA methylase (RmtB) | YP_001816610·1 | 41 | 96 |
| F06RAGE_8 | 16S rRNA methylase | YP_001816610·1 | 43 | 96 |
| F06RAGE_19 | 16S rRNA methylase | ADW66527·1 | 44 | 88 |
| F11GE_5 | 16S rRNA methylase | ADW66527·1 | 98 | 79 |
| F11GE_8 | 16S rRNA methylase | ABJ53409·1 | 51 | 96 |
| F11GE_10 | 16S rRNA methylase (MraW) | YP_003831185·1 | 63 | 98 |
| F11GE_11 | 16S rRNA methylase | ADW66527·1 | 98 | 58 |
| F19GE_5 | 16S rRNA methylase | ADW66527·1 | 99 | 99 |
| F19GE_1 | 16S rRNA methylase (MraW) | ZP_05272723·1 | 56 | 98 |
| F19GE_3 | 16S rRNA methylase | AFC75738·1 | 42 | 96 |
| F33GE_25 | 16S rRNA methylase | AFC75738·1 | 42 | 96 |

## Table S7: Selected chloramphenicol resistance genes

| | Age group | Annotation | Mechanism | Syntenic with | Top hit (nr) | % ID | % coverage |
|---|---|---|---|---|---|---|---|
| F04CH_25 | >12mo | Group A chloramphenicol acetyltransferase | Enzymatic modification | TraG conjugative transfer protein | ACT97523 | 100 | 100 |
| F18CH_6 | >12mo | Group A chloramphenicol acetyltransferase | Enzymatic modification | MobA/MobL mobilization protein | YP_001144149·1 | 100 | 100 |
| F07CH_1 | <=12mo | Group A chloramphenicol acetyltransferase | Enzymatic modification | rRNA methyltransfe rase | ZP_09342139·1 | 55 | 98 |
| F30CH_26 | <=12mo | Group A chloramphenicol acetyltransferase | Enzymatic modification | MobA mobilization protein | YP_001144149·1 | 97 | 99 |
| F29CH_39 | <=12mo | Major facilitator superfamily chloramphenicol exporter | Efflux | Transposase | NP_862236·1 | 100 | 100 |
| F16_RACH _1 | >12mo | Major facilitator superfamily multidrug exporter | Efflux | -- | ZP_07140420·1 | 99 | 100 |
| F30CH_34 | <=12mo | MarA (multiple antibiotic resistance) protein | Transcriptional regulator | MarC, MarR, MarB | YP_852664·1 | 99 | 100 |
| F22CH_4 | >12mo | MarA (multiple antibiotic resistance) protein | Transcriptional regulator | MarC, MarR | ZP_06990264·1 | 99 | 97 |
| F22CH_15 | >12mo | SoxS regulatory protein | Transcriptional regulator | SoxR | NP_290695·1 | 100 | 100 |
| F09CH_1 | <=12mo | SoxS regulatory protein | Transcriptional regulator | SoxR | YP_003366907·1 | 99 | 100 |
| F18CH_14 | >12mo | Rob regulatory protein | Transcriptional regulator | -- | NP_757329·1 | 100 | 100 |
| F30CH_16 | <=12mo | Rob regulatory protein | Transcriptional regulator | -- | NP_291009·1 | 100 | 100 |

## Table S8: Selected tetracycline resistance genes.

| Contig Name | Age group | Annotation | Mechanism | Syntenic with | Top hit (nr) | % ID | % coverage |
|---|---|---|---|---|---|---|---|
| F04TE_27 | >12mo | TetO ribosomal protection protein | Target protection | -- | ZP_02086796·1 | 99 | 100 |
| F15TE_1 | <=12mo | TetO ribosomal protection protein | Target protection | -- | ZP_01962904·1 | 99 | 99 |
| F29TE_12 | <=12mo | TetQ ribosomal protection protein | Target protection | RteA tetracycline response protein | ZP_02030811·1 | 99 | 99 |
| F11TE_2 | >12mo | TetQ ribosomal protection protein | Target protection | -- | ZP_04538372·1 | 99 | 99 |
| F29TE_11 | <=12mo | Tet32 ribosomal protection protein | Target protection | Tet40 tetracycline exporter | CAC41371·2 | 99 | 100 |
| F16RATE_10 | >12mo | Tet32 ribosomal protection protein | Target protection | -- | ZP_07958839·1 | 97 | 100 |
| F05TE_13 | <=12mo | TetW ribosomal protection protein | Target protection | TraG family conjugative transfer protein | ZP_03708733·1 | 99 | 100 |
| F22TE_30 | >12mo | TetM ribosomal protection protein | Target protection | -- | ZP_07771063·1 | 100 | 100 |
| F30TE_32 | <=12mo | TetM ribosomal protection protein | Target protection | TetL, plasmid recombinase | ZP_07467524·1 | 99 | 100 |
| F30TE_15 | <=12mo | TetL major facilitator superfamily tetracycline exporter | Efflux | TetM, plasmid recombinase | ZP_07467523·1 | 100 | 100 |
| F30TE_90 | <=12mo | TetA major facilitator superfamily tetracycline exporter | Efflux | -- | YP_003829179·1 | 100 | 100 |
| F29TE_11 | <=12mo | Tet40 major facilitator superfamily tetracycline exporter | Efflux | Tet32 ribosomal protection protein | ACI02010·1 | 100 | 100 |
| F21TE_4 | >12mo | TetV major facilitator superfamily tetracycline exporter | Efflux | -- | ZP_08129301·1 | 65 | 95 |
| F24TE_4 | >12mo | TetX1 flavin-dependent monooxygenase | Enzymatic modification | TetX2 mono-oxygenase | ZP_07041823·1 | 99 | 94 |
| F33_TE_8 | >12mo | TetX2 flavin-dependent monooxygenase | Enzymatic modification | TetX1 mono-oxygenase | ZP_07812201·1 | 99 | 100 |
| F18_TE_45 | >12mo | Multi Antimicrobial Extrusion (MATE) efflux family protein | Efflux | -- | CBL14746·1 | 97 | 100 |
| F22TE_29 | >12mo | Multi Antimicrobial Extrusion (MATE) efflux family protein | Efflux | -- | CBL19515·1 | 98 | 100 |

## Table S9: Selected tigecycline resistance genes.

| Contig Name | Age group | Annotation | Mechanism | Syntenic with | Top hit (nr) | % ID | % coverage |
|---|---|---|---|---|---|---|---|
| F15TG_1 | <=12mo | TetX1 flavin-dependent monooxygenase | Enzymatic modification | TetX2 monooxygenase | ZP_07041823·1 | 100 | 100 |
| F15TG_1 | <=12mo | TetX2 flavin-dependent monooxygenase | Enzymatic modification | TetX1 monooxygenase | ZP_07000543·1 | 99 | 100 |
| F15TG_1 | <=12mo | TetX2 flavin-dependent monooxygenase | Enzymatic modification | Aminoglycoside adenylyltransferase, mobilization protein BmgA | ZP_07000543·1 | 98 | 100 |

## Table S10: Selected trimethoprim resistance genes

| Contig Name | Age group | Annotation | Mechanism | Syntenic with | Top hit (nr) | % ID | % coverage |
|---|---|---|---|---|---|---|---|
| F33TR_19 | >12mo | Dfra22 Group A drug-insensitive dihydrofolate reductase | Target modification | integrase | ZP_07853644·1 | 100 | 100 |
| F04TR_128 | >12mo | Dfra26 Group A drug-insensitive dihydrofolate reductase | Target modification | transposase | ZP_02424783·1 | 100 | 100 |
| F12TR_21 | >12mo | Dfra12 Group A drug-insensitive dihydrofolate reductase | Target modification | integrase | ZP_05660529·1 | 100 | 100 |
| F21TR_27 | >12mo | Dfra24 Group A drug-insensitive dihydrofolate reductase | Target modification | thymidylate synthase, transposase | ZP_04856912·1 | 100 | 99 |
| F24TR_43 | >12mo | Dfra12 Group A drug-insensitive dihydrofolate reductase | Target modification | integrase, aminoglycoside adenylyltransferase | ZP_05660529·1 | 98 | 100 |
| F34TR_22 | <=12mo | Dfra22 Group A drug-insensitive dihydrofolate reductase | Target modification | MobA mobilization protein | ZP_07853644·1 | 96 | 62 |
| F09TR_2 | <=12mo | Dfra26 Group A drug-insensitive dihydrofolate reductase | Target modification | Permease | ZP_04742241·2 | 100 | 100 |
| F30TR_5 | <=12mo | Dfra12 Group A drug-insensitive dihydrofolate reductase | Target modification | transposase, GroEl integrase | YP_005221020·1 | 94 | 100 |
| F07TR_16 | <=12mo | Dfra25 Group A drug-insensitive dihydrofolate reductase | Target modification | thymidylate synthase | ZP_01961549·1 | 100 | 100 |
| F05TR_9 | <=12mo | Dfra20 Group A drug-insensitive dihydrofolate reductase | Target modification | thymidylate synthase, ABC transporter | CBL26888·1 | 99 | 100 |
| F15TR_18 | <=12mo | Dfra22 Group A drug-insensitive dihydrofolate reductase | Target modification | multi antimicrobial extrusion (MATE) efflux protein | ZP_07853644·1 | 100 | 100 |
| F11TR_35 | >12mo | Dfra22 Group A drug-insensitive dihydrofolate reductase | Target modification | thymidylate synthase, rRNA methyltransferase, multi antimicrobial extrusion (MATE) efflux protein, | ZP_01771126·1 | 92 | 100 |

## Table S11: Selected Trimethoprim-Sulfamethoxazole Resistance Genes

| Contig Name | Age group | Annotation | Mechanism | Syntenic with | Top hit (nr) | % ID | % coverage |
|---|---|---|---|---|---|---|---|
| F30TRSX_13 | <=12mo | Dfra12 Group A drug-insensitive dihydrofolate reductase | Target modification | aminoglycoside adenylyltransferase | NP_775043·1 | 100 | 100 |
| F34TRSX_12 | <=12mo | Dfra22 Group A drug-insensitive dihydrofolate reductase | Target modification | transposase | ZP_07853644·1 | 100 | 100 |
| F06RATRSX_138 | >12mo | Dfra20 Group A drug-insensitive dihydrofolate reductase | Target modification | thymidylate synthase, OrfB transposase | ZP_02438861·1 | 100 | 100 |
| F24TRSX_31 | >12mo | Dfra22 Group A drug-insensitive dihydrofolate reductase | Target modification | thymidylate synthase, transposase | ZP_04062765·1 | 100 | 100 |
| F11TRSX_80 | >12mo | Dfra22 Group A drug-insensitive dihydrofolate reductase | Target modification | Multi Antimicrobial Extrusion (MATE) efflux protein, thymidylate synthase, rRNA methylase | ZP_01771126·1 | 92 | 100 |
| F19TRSX_23 | >12mo | Dfra20 Group A drug-insensitive dihydrofolate reductase | Target modification | Macrolide-Lincosamide-Streptogramin ABC transporter, thymidylate synthase, TraE conjugal transfer protein | ZP_05270180·1 | 45 | 98 |
| F7TRSX_24 | <=12mo | Dfra20 Group A drug-insensitive dihydrofolate reductase | Target modification | ABC transporter | ZP_02042594·1 | 99 | 100 |

## Table S12: Selected Cycloserine Resistance Genes

| Contig Name | Age group | Annotation | Mechanism | Syntenic with | Top hit (nr) | % ID | % coverage |
|---|---|---|---|---|---|---|---|
| F12CY_91 | >12mo | D-ala-D-ala ligase | Target modification | UDP-N-acetylmuramoyl tripeptide D-ala-D-ala ligase | ACT97573·1 | 81 | 100 |
| F15CY_51 | <=12mo | D-ala-D-ala ligase | Target modification | ABC transporter | ZP_02027989·1 | 99 | 100 |
| F30CY_50 | <=12mo | D-ala-D-ala ligase | Target modification | Penicillin Binding Protein 2B, Recombination protein RecR | ZP_07466157·1 | 99 | 100 |
| F16CY_74 | >12mo | D-ala-D-ala ligase | Target modification | Transposase, UDP-N-acetylmuramoyl tripeptide D-ala-D-ala ligase | ZP_02429741·1 | 100 | 100 |
| F18CY_25 | >12mo | D-ala-D-ala ligase | Target modification | UDP-N-acetylmuramoyl tripeptide D-ala-D-ala ligase, RecG recombination protein | ZP_02025407·1 | 99 | 99 |

1. Goodman AL*, et al.* (2011) Extensive personal human gut microbiota culture collections characterized and manipulated in gnotobiotic mice. *Proc Natl Acad Sci U S A* 108(15):6252-6257.
2. Sommer MO, Dantas G, & Church GM (2009) Functional characterization of the antibiotic resistance reservoir in the human microflora. *Science* 325(5944):1128-1131.
3. Lutz R & Bujard H (1997) Independent and tight regulation of transcriptional units in Escherichia coli via the LacR/O, the TetR/O and AraC/I1-I2 regulatory elements. *Nucleic Acids Res* 25(6):1203-1210.
4. Zerbino DR & Birney E (2008) Velvet: algorithms for de novo short read assembly using de Bruijn graphs. *Genome Res* 18(5):821-829.
5. de la Bastide M & McCombie WR (2007) Assembling genomic DNA sequences with PHRAP. *Curr Protoc Bioinformatics* Chapter 11:Unit11.14.
6. Tatusov RL, Galperin MY, Natale DA, & Koonin EV (2000) The COG database: a tool for genome-scale analysis of protein functions and evolution. *Nucleic Acids Res* 28(1):33-36.
7. Forsberg KJ*, et al.* (2012) The shared antibiotic resistome of soil bacteria and human pathogens. *Science* In Press.
8. Edgar RC (2004) MUSCLE: multiple sequence alignment with high accuracy and high throughput. *Nucleic Acids Res* 32(5):1792-1797.
9. Price MN, Dehal PS, & Arkin AP (2010) FastTree 2--approximately maximum-likelihood trees for large alignments. *PLoS One* 5(3):e9490.
10. CLSI (2009) *Methods for Dilution Antimicrobial Susceptibility Tests for Bacteria That Grow Aerobically; Approved Standard-- Eighth Edition* (Clinical and Laboratory Standards Institute, Wayne, PA).
